# PIRACY: An Optimized Pipeline for Functional Connectivity Analysis in the Rat Brain

**DOI:** 10.3389/fnins.2021.602170

**Published:** 2021-03-26

**Authors:** Yujian Diao, Ting Yin, Rolf Gruetter, Ileana O. Jelescu

**Affiliations:** ^1^Animal Imaging and Technology, EPFL, Lausanne, Switzerland; ^2^CIBM Center for Biomedical Imaging, Lausanne, Switzerland; ^3^Laboratoire d’Imagerie Fonctionnelle et Métabolique, EPFL, Lausanne, Switzerland

**Keywords:** data processing pipeline, global signal regression, ICA, resting state – fMRI, functional connectivity, rat – brain, denoising

## Abstract

Resting state functional MRI (rs-fMRI) is a widespread and powerful tool for investigating functional connectivity (FC) and brain disorders. However, FC analysis can be seriously affected by random and structured noise from non-neural sources, such as physiology. Thus, it is essential to first reduce thermal noise and then correctly identify and remove non-neural artifacts from rs-fMRI signals through optimized data processing methods. However, existing tools that correct for these effects have been developed for human brain and are not readily transposable to rat data. Therefore, the aim of the present study was to establish a data processing pipeline that can robustly remove random and structured noise from rat rs-fMRI data. It includes a novel denoising approach based on the Marchenko-Pastur Principal Component Analysis (MP-PCA) method, FMRIB’s ICA-based Xnoiseifier (FIX) for automatic artifact classification and cleaning, and global signal regression (GSR). Our results show that: (I) MP-PCA denoising substantially improves the temporal signal-to-noise ratio, (II) the pre-trained FIX classifier achieves a high accuracy in artifact classification, and (III) both independent component analysis (ICA) cleaning and GSR are essential steps in correcting for possible artifacts and minimizing the within-group variability in control animals while maintaining typical connectivity patterns. Reduced within-group variability also facilitates the exploration of potential between-group FC changes, as illustrated here in a rat model of sporadic Alzheimer’s disease.

## Introduction

Resting state functional MRI (rs-fMRI) based on spontaneous low-frequency fluctuations in the blood oxygen level dependent (BOLD) signal in the resting brain is a widely used non-invasive tool for studying intrinsic functional organization in health and disease (Fox and Raichle, 2007; Fornito and Bullmore, 2010). By examining spatio-temporal correlations of the BOLD signal between distinct brain regions, known as functional connectivity (FC), this technique is capable of revealing large-scale resting state networks (RSNs) (Biswal et al., 1995; Damoiseaux et al., 2006; Buckner et al., 2013). Nowadays, rs-fMRI has become an increasingly important translational neuroimaging tool for understanding neurological and psychiatric diseases and for developing treatments, with rapidly growing applications not only in human research but also in rodent models of disease (Fox and Greicius, 2010; Bajic et al., 2017).

However, the BOLD signal is contaminated by multiple physiological and non-physiological sources of noise, such as respiratory and cardiac cycles, thermal noise, changes in blood pressure, and head motion (Kruger and Glover, 2001; Birn, 2012; Van Dijk et al., 2012; Murphy et al., 2013). These non-neuronal sources can severely affect rs-fMRI time series and thereby confound the connectivity analysis (Cole et al., 2010; Power et al., 2014). Therefore, a robust pre-processing pipeline is required to extract the neuronal component of the BOLD signal and minimize the contribution of such noise sources. Furthermore, existing tools that correct for the effect of non-neuronal sources are mostly tailored for human rs-fMRI data and are not readily transposable, or even applicable, to rodent data. Dedicated pipelines for rodent rs-fMRI processing are just starting to emerge (Zerbi et al., 2015; Bajic et al., 2017).

For example, signal fluctuations resulting from respiratory and cardiac cycles can be accounted for by explicitly recording the physiology and modeling these external confounds as regressors (Birn et al., 2006; Kasper et al., 2017). While physiological recordings in rodents are possible, they typically involve dedicated hardware and invasive procedures, making them experimentally difficult. However, although cardiac and respiratory frequencies in rodents are much higher than those of the resting-state BOLD fluctuations, depending on the temporal resolution of the acquisition, they can alias into the band of interest (typically 0.01–0.3 Hz) and corrupt the analysis. Two complementary approaches are, therefore, suitable to mitigate the impact of physiological noise in rodent rs-fMRI.

One approach is the removal of global signal defined as the mean time series averaged over all voxels within the brain by including the global signal as a nuisance regressor in General Linear Model (GLM) analyses, which is referred to as global signal regression (GSR) (Liu et al., 2017). However, the use of GSR has been one of the most controversial topics in human rs-fMRI connectivity studies (Liu et al., 2017; Murphy and Fox, 2017). On one hand, GSR is known to introduce spurious negative correlations (Murphy et al., 2009) and cause spatial bias on connectivity measures (Saad et al., 2012). On the other hand, prior studies have shown that GSR can enhance the detection of significant FC and improve spatial specificity of positive correlations (Fox et al., 2009). Most importantly for rodent studies, GSR can also mitigate confounds related to motion and physiological processes (Power et al., 2015; Aquino et al., 2019).

Another commonly used data-driven approach that identifies various physiological noise components directly from the fMRI data itself is single-level independent component analysis (ICA) (McKeown et al., 2003; Griffanti et al., 2014; Bajic et al., 2017; Caballero-Gaudes and Reynolds, 2017). The ICA method is also confronted by several issues including model order selection (i.e., the number of components) (Kuang et al., 2018) and the identification of artifactual components, which is a manually tedious step (Wang and Li, 2015), especially for a high order model. Notably, a machine-learning approach for automatic artifact component classification based on FMRIB’s ICA-based Xnoiseifier (FIX) (Salimi-Khorshidi et al., 2014) has been proposed to replace manual classification. The FIX auto-classifier applied in human and mice rs-fMRI studies has yielded promising results with a high accuracy in artifact identification (Griffanti et al., 2014, 2015; Zerbi et al., 2015). However, the success of FIX classification relies on a proper pre-training on study-specific datasets.

Therefore, the aim of the present study was to propose and evaluate a data processing pipeline for rat rs-fMRI that minimizes intra-group variability and maximizes between-group differences in whole-brain FC. In this pipeline, we reduced structural noise by combining single-session ICA cleaning and GSR. For ICA cleaning, we built and used a dedicated FIX classifier for rats. Furthermore, we enhanced the sensitivity to the BOLD fluctuations by first increasing dramatically the temporal signal-to-noise ratio (tSNR) of the data. For the purpose of stochastic (thermal) noise removal, we employed a novel method based on Marchenko-Pastur Principal Component Analysis (MP-PCA). MP-PCA denoising was recently introduced for diffusion MRI and is a model-free method that exploits redundancy in MRI series (Veraart et al., 2016), which has shown great potential for improving the SNR in other MRI techniques as well (Does et al., 2019; Ades-Aron et al., 2020).

## Materials and Methods

### Animal Preparation and Anesthesia

All experiments were approved by the local Service for Veterinary Affairs. Male Wistar rats (236 ± 11 g) underwent a bilateral intracerebroventricular (icv) injection of either streptozotocin (3 mg/kg, STZ group) or buffer (control group). When delivered exclusively to the brain, streptozotocin induces impaired brain glucose metabolism and is used as a model of sporadic Alzheimer’s disease (AD) (Lester-Coll et al., 2006; Knezovic et al., 2015; Grieb, 2016).

**FIGURE 1:**
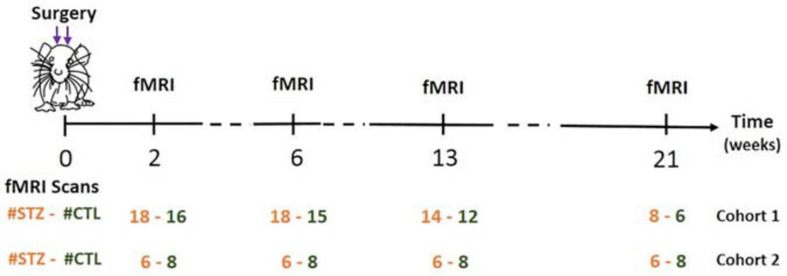
Timeline of experiments. Two fMRI runs were acquired per rat for each experiment. Figure adapted from Tristão Pereira et al. (2021), NeuroImage 2021, with permission.

As a result of system upgrade, the fMRI data were acquired on two rat cohorts (1/2) on different MRI consoles (Varian/Bruker). We refer to animals scanned on the Varian system as Cohort 1 (*N* = 17 rats) and to animals scanned on the Bruker system as Cohort 2 (*N* = 7 rats). Each cohort comprised animals from both groups: Cohort 1 (CTL/STZ, *N* = 8/9 rats) and Cohort 2 (CTL/STZ, *N* = 4/3 rats), which were scanned at 2, 6, 13, and 21 weeks after the surgery (Figure 1). Rats were anesthetized using 2% isoflurane in a mixture of O_2_ and air (O_2_/air: 30/70) during the initial setup and promptly switched to medetomidine sedation delivered through a subcutaneous catheter in the back (bolus: 0.1 mg/kg, perfusion: 0.1 mg/kg/h) as previously described (Reynaud et al., 2019). Medetomidine preserves neural activity and vascular response better than isoflurane (Weber et al., 2006; Pawela et al., 2009; Grandjean et al., 2014). The rat head was fixed using a homemade holder with a bite bar and ear bars to minimize the head motion, and body temperature and breathing rate were continuously monitored. At the end of the scanning sessions, rats were woken up with an intra-muscular injection of antagonist atipamezole (0.5 mg/kg) and returned to their cages.

### MRI Acquisition

Magnetic resonance imaging experiments were conducted on a 14.1 T small animal scanner with two different consoles: Varian system (Varian Inc.) equipped with 400 mT/m gradients (Cohort 1) and Bruker system (Ettlingen, Germany) equipped with 1 T/m gradients (Cohort 2), both using an in-house built quadrature surface transceiver. The acquisition parameters were the same for the two cohorts.

An anatomical reference scan was acquired using a fast spin-echo multi-slice sequence with the following parameters: TE/TR = 10.17/3000 ms, ETL = 4, TE_eff_ = 10.17 ms, field of view (FOV) = 19.2 mm × 19.2 mm, matrix = 128 × 128, in-plane resolution = 150 μm × 150 μm, number of slices = 30, and thickness = 0.5 mm. Before running the fMRI sequence, anesthesia was switched from isoflurane to medetomidine. The fMRI acquisitions were started after a fixed duration (∼1 h) since the switch from isoflurane to medetomidine to minimize between-animal anesthesia-related confounds. Rs-fMRI data were acquired using a two-shot gradient-echo echo-planar imaging (EPI) sequence as follows: TE = 10 ms, TR = 800 ms, TR_vol_ = 1.6 s, FOV = 23 mm × 23 mm, matrix = 64 × 64, in-plane resolution = 360 μm × 360 μm, 8 slices, thickness = 1.12 mm, and 370 repetitions (∼10 min). Two fMRI runs were acquired for each rat. A short scan with 10 repetitions and reversed phase-encoding direction was also acquired to correct for EPI-related geometric distortions.

### FMRI Data Pre-processing

Our data processing pipeline was developed based on the data of Cohort 1.

Anatomical and fMRI images were first skull-stripped separately using BET (Brain Extraction Tool; FSL)^1^ (Smith, 2002), and fMRI time series were denoised using MP-PCA with a 5 × 5 × 5 voxel sliding kernel (Veraart et al., 2016). The quality of MP-PCA denoising was assessed by inspecting the normality of the residuals (original – denoised) and the tSNR changes before and after denoising. Specifically, the normality of the residuals was tested by the linearity of the relationship between the natural logarithm of the residual distribution probability and the squares of multiple residual standard deviation. Then, the datasets went through EPI distortion correction using FSL’s topup (Smith et al., 2004), slice-timing correction (Henson et al., 1999; Calhoun et al., 2000; Sladky et al., 2011), and spatial smoothing (Gaussian kernel: 0.36 mm × 0.36 mm × 1 mm). Corrected fMRI images were registered to the Waxholm Space Atlas of the rat brain^2^ using linear and non-linear registration in ANTs (Avants et al., 2008). Highly parcelated atlas labels were grouped to yield larger, anatomically consistent, labels [e.g., all sub-regions pertaining to the anterior cingulate cortex (ACC) were grouped into one ACC label], and 28 atlas-defined regions of interest (ROIs, 14 per hemisphere) were thus automatically segmented. The brain mask extracted based on EPI images was used to mask atlas labels in fMRI space such that only regions with sufficient signal were included and areas of drop-out were excluded.

Finally, single-session ICA was performed on fMRI time courses using FSL’s MELODIC (Beckmann and Smith, 2004) with high-pass temporal filtering (*f* > 0.01 Hz) and 40 independent components (ICs).

### FIX Training

Datasets of Cohort 1 were randomly split into two groups: a training dataset for FIX (*n* = 49) and a test dataset (*n* = 58). The ICA components in the training dataset were manually classified to signal or artifact, which was mainly based on thresholded spatial maps because ICA is theoretically more robust in the spatial than in the temporal domain (Smith et al., 2012; Salimi-Khorshidi et al., 2014). Generally speaking, spatial maps of signal components should contain a low number of anatomically consistent clusters, whereas artifactual components typically have either very large clusters covering brain slices or very small and scattered clusters (Griffanti et al., 2017). Here, we chose an “aggressive” artifact removal (Griffanti et al., 2014) in the training dataset in order to give the trained classifier a margin to be conservative or aggressive *via* adjusting the threshold fed to it (small thresholds make it conservative).

The performance of the trained classifier in detecting artifactual components was evaluated on the test dataset by comparing the automatic classification of artifactual components with the manual classification. The classification accuracy was characterized in terms of “recall” and “precision” (Powers, 2011), which are defined as the percentage of the correctly predicted artifact components in all actual artifact components and the percentage of the correctly predicted artifact components in all predicted artifact components, respectively.

### Network Analysis and GSR

After ICA decomposition and classification, the artifactual components were regressed out of the 4D pre-processed datasets to obtain “cleaned” rs-fMRI datasets. The cleaned data were used to compute ROI-to-ROI FC by calculating correlation coefficients between the ROI-averaged time series of the 28 atlas-defined ROIs, resulting in a 28 × 28 FC matrix for each rat.

For FIX, we relied on spatial maps rather than time courses to evaluate artifactual components. In several cases, components displayed sensible spatial distribution to represent a RSN, but the power spectrum showed a peak at a frequency that could be attributed to breathing (Supplementary Figure 1) (the breathing rate was recorded for each run and its aliased frequency within our 0.01–0.31 Hz band was calculated). These non-neuronal sources that could not be cleaned using FIX also contribute to the global signal. To mitigate their effect, we used the partial correlation of ROI-to-ROI time courses to build FC matrices, with the global signal as the controlling variable. For every pair of ROIs, the partial correlation was implemented by measuring the correlation between their time-series residuals, after each having been adjusted by the GSR (Smith et al., 2011).

Finally, statistical comparisons of FC between the STZ and CTL groups at each timepoint were performed using NBS (Zalesky et al., 2010) to identify network connections that showed significant between-group difference. Specifically, NBS uses one-tailed two-sample *t*-test to detect differences in group-averaged FC between the two groups. Thereby, two contrasts (*STZ* > *CTL* and *STZ* < *CTL*) were tested separately. A *t*-statistic threshold was chosen on the basis of medium-to-large sizes of the subnetwork comprised connections with their *t*-statistic above the threshold (Tsurugizawa et al., 2019) as well as the underlying *p*-values. Here, we chose 2.2 as the *t*-statistic threshold. Results based on other thresholds are provided as Supplementary Figures 2–4. Significance (*p* ≤ 0.05) was tested after family wise error rate (FWER) correction using non-parametric permutation (*N* = 5000).

**FIGURE 2:**
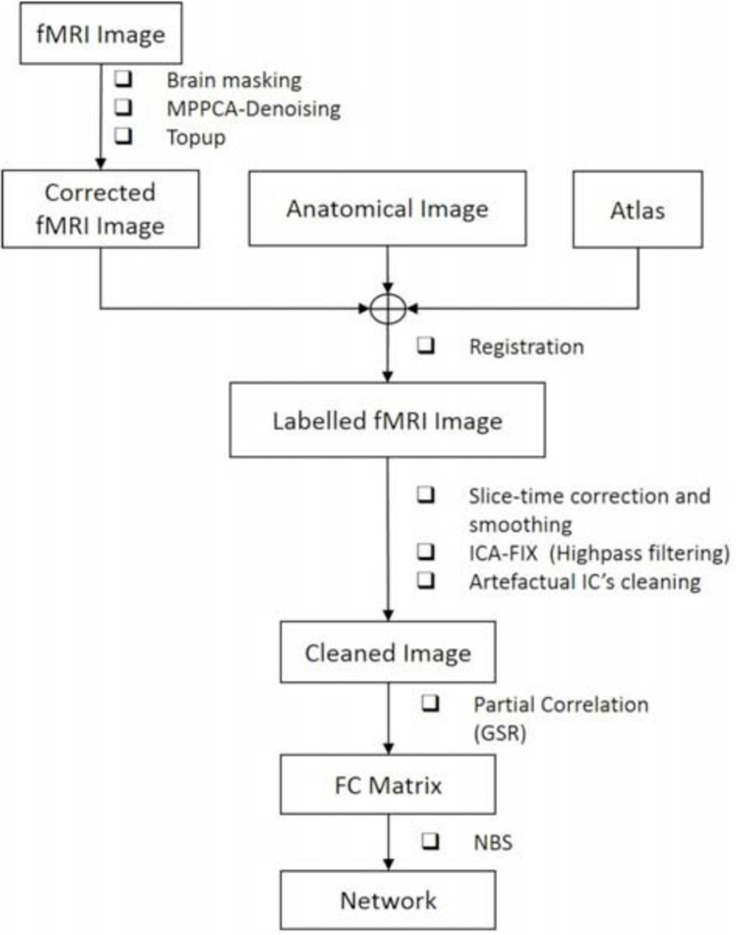
The proposed pipeline for rs-fMRI data processing.

The full data processing pipeline is illustrated in Figure 2.

### Pipeline Evaluation on Cohort 1

To evaluate the rs-fMRI data processing pipeline including MP-PCA denoising (DN), slice-timing correction (SC), spatial smoothing (SM), high-pass filtering (HP), ICA-FIX cleaning (CL), and GSR in terms of consistency of within-group FC in the healthy CTL group and in terms of between-group difference, we compared results of the PIRACY (Pipeline for Rat Connectivity) pipeline (DN + CL + GSR, pipeline G) with other processing approaches depending on the presence of DN, CL, and GSR in addition to the baseline pipeline (SC + SM + HP, pipeline A), namely, GSR (pipeline B), CL + GSR (pipeline C), DN (pipeline D), DN + CL (pipeline E), and DN + GSR (pipeline F) in Cohort 1, shown in Table 1.

**TABLE 1:** The seven data processing pipelines and methods they include (“×” – including, “∘” – excluding).

Pipelines	MP-PCA-denoising (DN)	ICA-FIX cleaning (CL)	Global signal regression (GSR)
A	**∘**	**∘**	**∘**
B	**∘**	**∘**	**×**
C	**∘**	**×**	**×**
D	**×**	**∘**	**∘**
E	**×**	**×**	**∘**
F	**×**	**∘**	**×**
G	**×**	**×**	**×**

*SC, SM, and HP are included in all of the seven pipelines.*

Based on the hypothesis that an optimal processing procedure should minimize the variability within the homogeneous group of healthy controls (Zerbi et al., 2015), the within-group variability was assessed by calculating the standard deviation of the Fisher *z*-transformed correlation coefficients of the FC matrices in the CTL group of healthy rats at each timepoint. In addition, the sensitivity to between-group differences was evaluated by comparing the significant difference in FC between the STZ and CTL groups at each timepoint.

### Group ICA Analysis

At each timepoint, group-level ICA was performed using FSL’s Melodic (Smith et al., 2004) on the pooled CTL and STZ datasets of Cohort 1 that were pre-processed using pipeline E (no GSR). Prior to group-level ICA in Melodic, all the rs-fMRI datasets from a given timepoint were registered to a common template using ANTs (Avants et al., 2008), and the template registration in Melodic was by-passed. Thirty group-level spatial ICs were extracted. The selection of IC number is a trade-off between biological detail (small and refined networks) and noise (too many components that can be supported by data quality) (Bajic et al., 2017). *N* = 30 or 40 components are common for rodent group ICA (Jonckers et al., 2011; Zerbi et al., 2015; Bajic et al., 2017). In our case, *N* = 30 had the advantage of yielding a similar number of nodes to the ROI-to-ROI FC analysis for the network analyses. Dual regression (Filippini et al., 2009) was used to estimate subject-specific time courses and associated spatial maps. Similar to the ROI-based FC analysis, ICA-based FC matrices were built by calculating correlation coefficients between all pairs of time courses (excluding artifact components) using FSLNets (Griffanti et al., 2014). Both full correlation (original Pearson’s correlation) and partial correlation with GSR (mean time course as regressor) were employed by FSLNets to evaluate the similarity between time courses. In the end, differences in FC between the STZ and CTL groups were tested using the NBS toolbox using the same parameters in the Network Analysis and GSR section.

### Pipeline Evaluation on Cohort 2

To test the robustness of the proposed pipeline, we further evaluated it on the independent datasets of Cohort 2. Similarly, we compared results of the PIRACY pipeline G (DN + CL + GSR) with three other processing pipelines: DN (D), DN + CL (E), and DN + GSR (F) in terms of the within-group variability for the control rats of Cohort 2. Then, we assessed the between-group differences in FC between the STZ and CTL groups on the pooled datasets of Cohorts 1 and 2.

## Results

**FIGURE 3:**
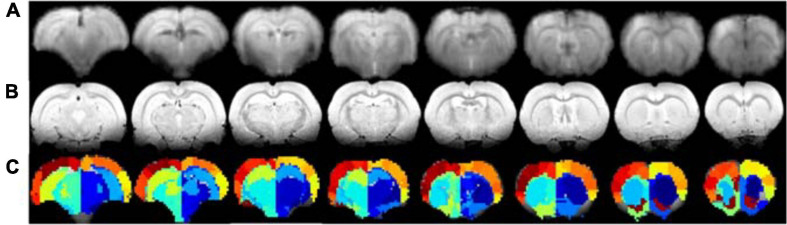
Example of rs-fMRI images of eight coronal slices **(A)**, matching anatomical MR images **(B)**, and atlas-based anatomical labels registered to the fMRI images **(C)**.

To experimentally evaluate the processing pipeline, a total of 109 rs-fMRI datasets (Cohort 1) were acquired from 17 rats at four timepoints ranging from 2 to 21 weeks (Figure 1). Two datasets with bad image quality were discarded. Figure 3 shows the MR images in one representative dataset including rs-fMRI images, matching anatomical reference, and the atlas-based anatomical labels registered to the fMRI images.

To assess the robustness of the PIRACY pipeline, an independent cohort with 56 rs-fMRI datasets was acquired from seven different rats at four timepoints ranging from 2 to 21 weeks (Figure 1).

### MP-PCA Denoising

**FIGURE 4:**
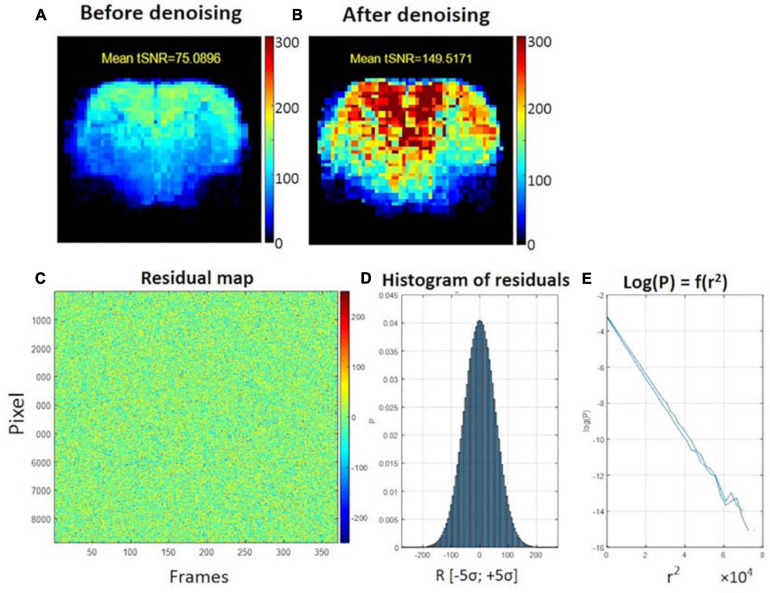
Example of temporal SNR maps, before **(A)** and after MP-PCA denoising **(B)**. The mean tSNR over the middle brain slice was improved dramatically from 75 to 146. The SNR profile is typical of a surface coil placed on top of the head, with higher sensitivity in the cortex. **(C)** Residuals map of all voxels within the brain mask (rows) and time frame (columns). **(D)** Histogram of residuals. **(E)** Normality test.

**FIGURE 5:**
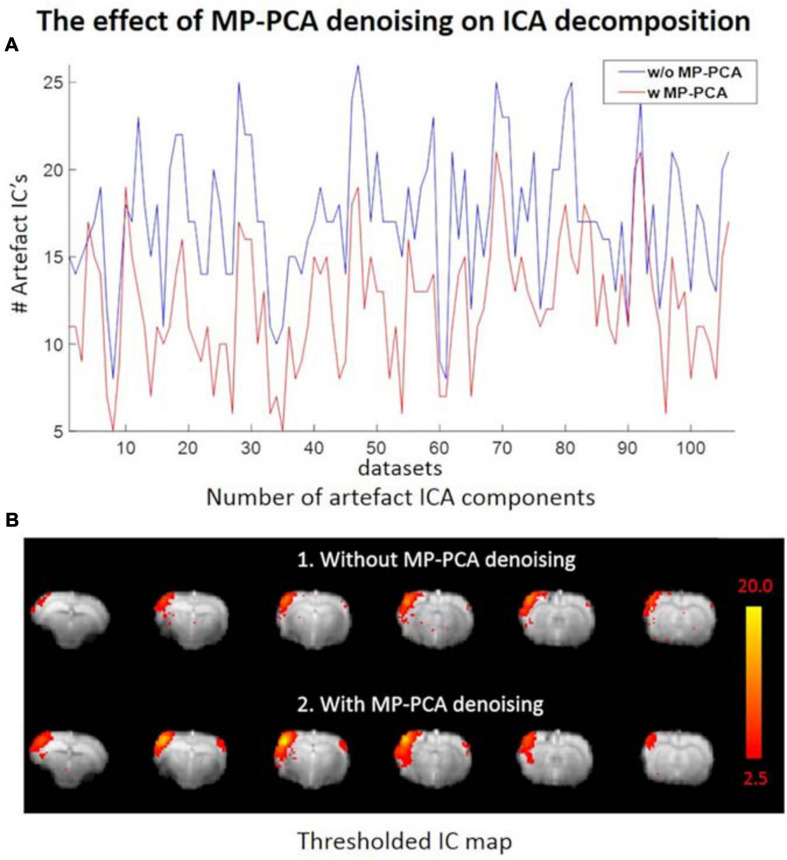
**(A)** Number of ICs classified as artifacts across all datasets without (blue) and with (red) prior MP-PCA denoising, respectively. MP-PCA denoising substantially reduced artifactual ICA components for most datasets. **(B)** Prior MP-PCA denoising improved the *z*-statistic for signal components. Thresholded IC maps in **(B)** were given by FSL’s MELODIC.

The average tSNR after MP-PCA denoising improved significantly for all the 107 datasets. Figure 4 shows an example of the average tSNR increase from 75 to 146 after MP-PCA denoising, residuals map, histogram, and the normality test. The linearity of log(P) = *f*(r^2^) confirms that the residuals are normally distributed and only Gaussian noise has been removed from the signal. Moreover, the number of components classified as artifacts decreased, whereas the *z*-statistic for signal components increased when MP-PCA denoising was applied prior to ICA decomposition (Figure 5).

### FIX Classification

Here, we preferred a lower order model to avoid overfitting (Li et al., 2007; Kuang et al., 2018), and we chose the number of ICs to be 40, which typically explained 90% of the variance. Notably, reaching 95% of explained variance would have required about 90 components, which would potentially cause over-splitting networks and making the classification more complicated.

The single-subject ICA was performed on each dataset with 40 components. In the training dataset (*n* = 49), 19.8 ± 4.7 components (50%) were classified as artifacts by hand. In the test dataset (*n* = 58), between 40 and 64% of components were classified as artifacts automatically by FIX depending on the threshold. More components could be recognized as artifacts by increasing the FIX threshold at the expense of lowering the classification precision due to more misclassification. Here, 45 might be an “optimal” threshold with overall 88% in recall and 90% in precision achieved (Table 2). In practice, we ran FIX twice with a low and high threshold, respectively. We then examined only the components differing between the two artifact lists given by the two FIX thresholds and manually restored the true noise components. This represented nonetheless a significant gain in processing time, as we only needed to classify one third of the ICA components by hand.

**TABLE 2:** FIX artifact classification accuracies at different thresholds.

*FIX threshold*	*20*	*30*	*40*	*45*	*50*	*60*	70
*Signal components (%)*	44.9	44.9	44.9	44.9	44.9	40.4	36.2
*Artifact components (%)*	39.6	43.9	49.4	52.7	55.1	59.6	63.8
*Unknown components (%)*	15.5	11.2	5.7	2.5	0	0	0
*FIX artifact recall (%)*	69.8	76.5	83.3	**87.8**	89.7	95.1	98.5
*FIX artifact precision (%)*	100	95.4	91.6	**90.2**	86.5	84.4	82.2

*Recall = correctly classified artifacts/all real artifacts, precision = correctly classified artifacts/all classified artifacts. As the threshold increases, both the percentage of artifacts detected by FIX and the recall increase, but the precision decreases. Namely, with increasing thresholds, more real artifact components are identified; however, signal components will also tend to be misclassified as artifacts. Bold font identifies an optimal threshold.*

### Comparison of Pipeline Performance

The seven data processing pipelines (A–G) were first compared based on resulting FC matrices of 28 atlas-based ROIs in Cohort 1.

**FIGURE 6:**
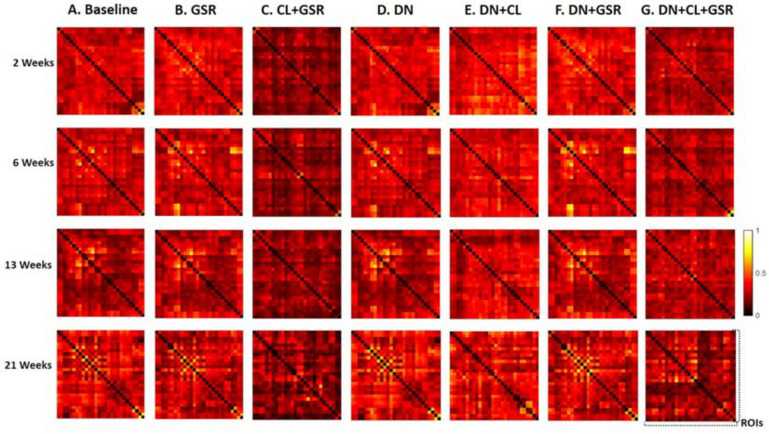
Standard deviation of the Fisher *z*-transformed correlation coefficient for functional connectivity in the CTL group of Cohort 1 at four different timepoints for the seven pipelines **(A–G)**. Pipelines including CL and GSR **(C,G)** obtained the minimal within-group variability in the homogeneous group of healthy controls for all timepoints, whereas other procedures excluding CL and/or GSR had higher variability.

**FIGURE 7:**
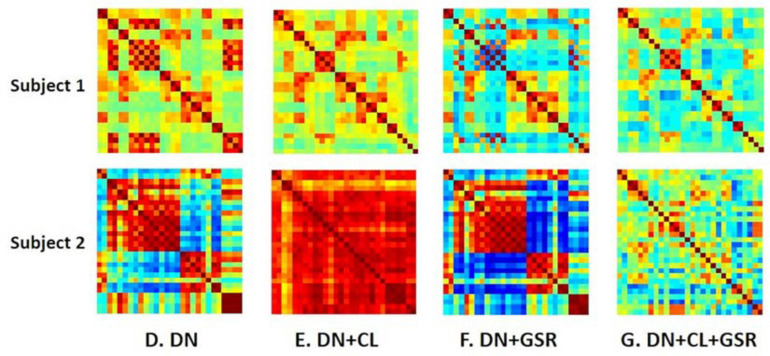
Example FC matrices of two control rats scanned at 2 weeks (Subjects 1 and 2) processed with four different pipelines **(D–G)**. For Subject 1, GSR alone (pipeline **F**) reinforces a widespread anti-correlation between the left and right hemispheres, manifest in a checkerboard pattern (odd/even rows and columns correspond to the left/right hemisphere ROIs, respectively) that was otherwise removed by ICA cleaning (pipeline **E**). For Subject 2, GSR alone does not attenuate very strong positive/negative correlation blocks spanning large portions of the brain, whereas ICA cleaning alone yields artificially high whole-brain connectivity. The combination of CL + GSR (pipeline **G**) mitigates these effects in both examples and yields more consistent between-subject connectivity matrices.

**FIGURE 8:**
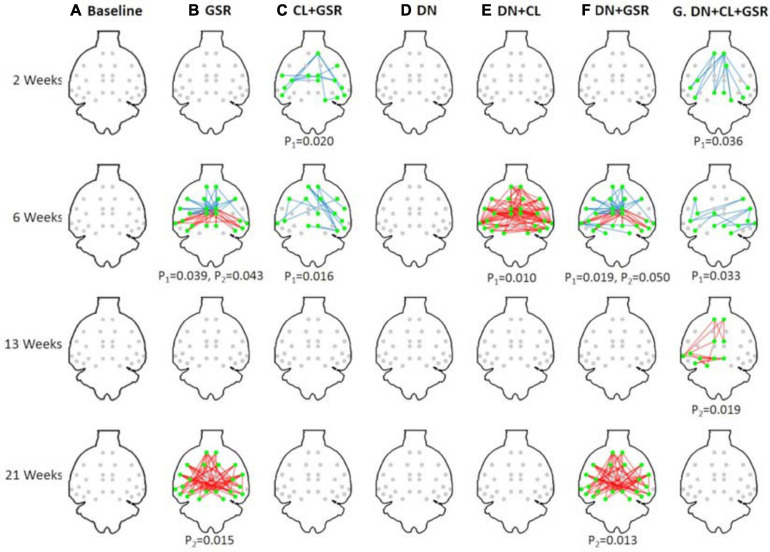
Significant differences in FC between the CTL and STZ groups of Cohort 1 at each timepoint (2, 6, 13, and 21 weeks) for each data processing approach under NBS threshold 2.2. No significant difference was found between the CTL and STZ groups for the baseline pipeline **(A)** and pipeline **(D)** (DN only), whereas significant differences were only found at 6 weeks for pipeline **(E)** (DN + CL, no GSR). Pipelines **(B,F)** (GSR, or DN + GSR) had similar results with between-group differences detected at 6 and 21 weeks. Pipeline **(G)** (DN + CL + GSR) most consistently yielded between-group differences across timepoints, whereas pipeline **(C)** (CL + GSR, no DN) only detected significant differences at two timepoints. Blue edges indicate group differences in contrast 1 (*STZ* > *CTL*), and red edges indicate group differences in contrast 2 (*STZ* < *CTL*). *p*_1_ and *p*_2_ are FWER corrected *p*-values for contrast 1 and contrast 2, respectively. Green nodes indicate ROIs involved in group differences.

Pipelines C and G that include both CL and GSR yielded lower within-group variability in the homogeneous group of healthy controls for all timepoints than other procedures excluding CL and/or GSR (Figure 6). The combination of CL + GSR also corrected for physiological artifacts that CL or GSR alone could not systematically address (Figure 7). Furthermore, our proposed pipeline G (DN + CL + GSR) yielded between-group differences most consistently at 2, 6, and 13 weeks, whereas pipeline C (without DN) only detected between-group differences at 2 and 6 weeks. No significant difference was found between the CTL and STZ groups for the baseline pipeline A and pipeline D (only DN). Pipeline E (DN + CL) exhibited numerous different edges at 6 weeks and none at the other timepoints, whereas pipelines B and F (GSR or DN + GSR) yielded numerous different edges at 6 and 21 weeks but none at 2 and 13 weeks (Figure 8).

### ROI-Based FC Analysis

**FIGURE 9:**
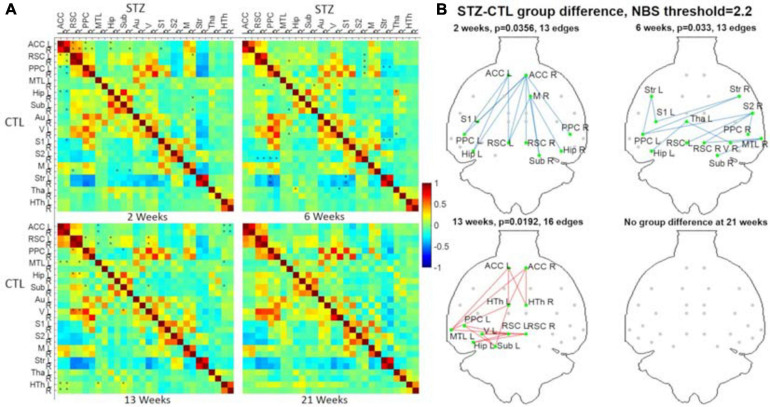
**(A)** Hybrid average FC matrices at each timepoint (top-right half: STZ, bottom-left half: CTL) based on the data of Cohort 1 processed by the optimized pipeline G. ^∗^: *p* < 0.05 (FWER corrected) at threshold of 2.2. **(B)** Graph networks at four timepoints. Blue/red edges and green nodes indicate connections with significant difference. The *p*-value for each network was given after FWER correction. ACC, anterior cingulate cortex; RSC, retrosplenial cortex; PPC, posterior parietal cortex; MTL, medial temporal lobe; Hip, hippocampus; Sub, subiculum; Au, auditory; V, visual; S1/S2, primary/secondary somatosensory; M, motor; Str, striatum; Tha, thalamus; HTh, hypothalamus; L/R, left/right. Figure adapted from Tristão Pereira et al. (2021), NeuroImage 2021, with permission.

To analyze group differences in connectivity obtained following the PIRACY pipeline G, a more detailed visualization is provided in Figure 9. The complete list of edges with significant between-group differences is provided as Supplementary Table 1. Nodes with altered connectivity in STZ animals were consistent with regions affected by AD and with previous findings on this animal model (Mayer et al., 1990; Shoham et al., 2003; Lester-Coll et al., 2006; Kraska et al., 2012; Grieb, 2016). At 2 weeks, increased positive connectivity of the ACC to the retrosplenial cortex (RSC) and decreased anti-correlations of the default mode network (DMN) including the ACC, RSC, posterior parietal cortex (PPC), and hippocampus (Hip/Sub) to the lateral cortical network (LCN) involving somatosensory (S1) as well as motor (M) were found. The 6-week timepoint showed widespread reduced anti-correlations between the DMN (including the RSC, PPC, Hip), somatosensory of the LCN, and striatum (Str). At 13 weeks, weaker positive correlations were found within the DMN involving not only the ACC, PPC, RSC, Hip, medial temporal lobe (MTL), and visual cortex (V) but also the hypothalamus (HTh) to the DMN.

### Group ICA

**FIGURE 10:**
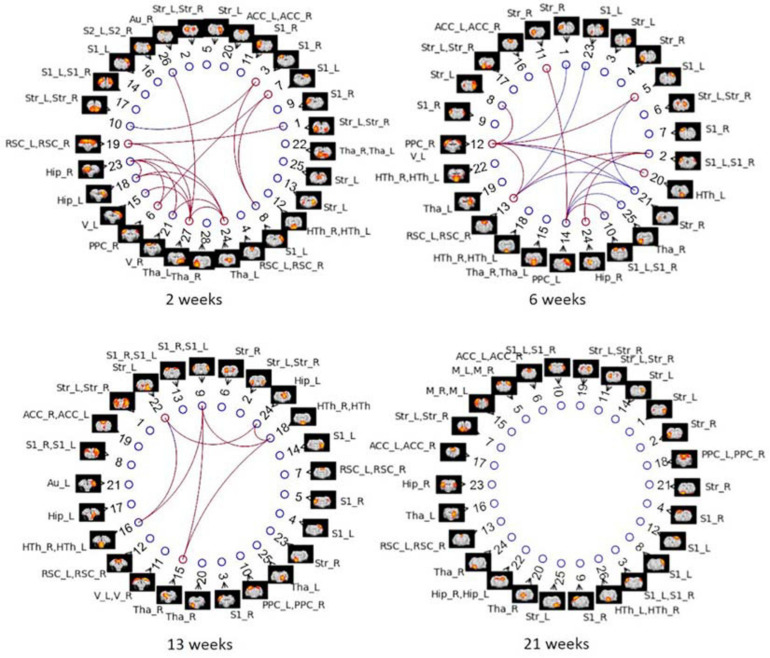
Between-group differences in ICA-based functional connectivity with GSR (partial correlation) at NBS threshold of 2.2 for each timepoint in Cohort 1. Colored edges display the existence of STZ–CTL difference in connections between ICs. Each IC is denoted by a spatial map and its IC number. The nodes of ICs are listed in an order based on its position in the brain (anterior to posterior). ROI labels are attached to every ICA component. Artifactual components were removed, and the IC number was reordered accordingly. Blue edges indicate group differences in contrast 1 (*STZ* > *CTL*), and red edges indicate group differences in contrast 2 (*STZ* < *CTL*).

Group ICA with 30 components was carried out on Cohort 1. Artifactual components were identified and removed, which resulted in 28, 25, 25, and 26 signal components left for the four timepoints, respectively. Figure 10 displays the significant between-group differences in partial correlations with GSR between ICA components. The complete list of edges with significant between-group differences is provided as Supplementary Table 2. Remarkably, group ICA analysis with GSR showed intergroup differences at three timepoints from 2 to 13 weeks, in agreement with differences found in ROI-based FC analysis using pipeline G. At 2 weeks, most changes were found in connections between the RSC, PPC, Hip, thalamus (Tha), and S1/2. At 6 weeks, there were alterations found in connectivity involving the PPC, Hip, RSC, S1/2, Str, as well as Tha. At 13 weeks, primary changes were detected in connections between the Hip, S1, Str, Tha, and HTh. Full correlation between ICA components–an equivalent to pipeline E: DN + CL–revealed significant differences at all timepoints (Supplementary Figure 5), in poor agreement with the ROI-based FC analysis using pipeline E, which only identified group differences at one timepoint (6 weeks). This inconsistency may indicate the importance of GSR in obtaining consistent intergroup differences.

### Pipeline Performance on Independent Datasets (Cohort 2)

**FIGURE 11:**
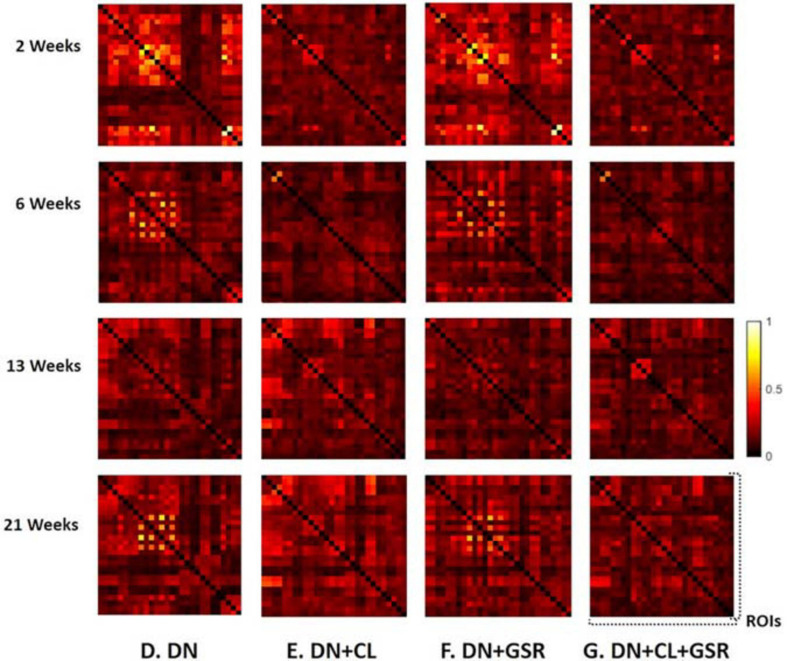
Standard deviation of the Fisher *z*-transformed correlation coefficient for functional connectivity in the CTL group of Cohort 2 at four different timepoints for four pipelines **(D–G)** all including DN. Pipeline **(G)** (DN + CL + GSR) yielded the minimal within-group variability in the homogeneous group of healthy controls for all timepoints, whereas other procedures excluding CL and/or GSR had higher variability.

**FIGURE 12:**
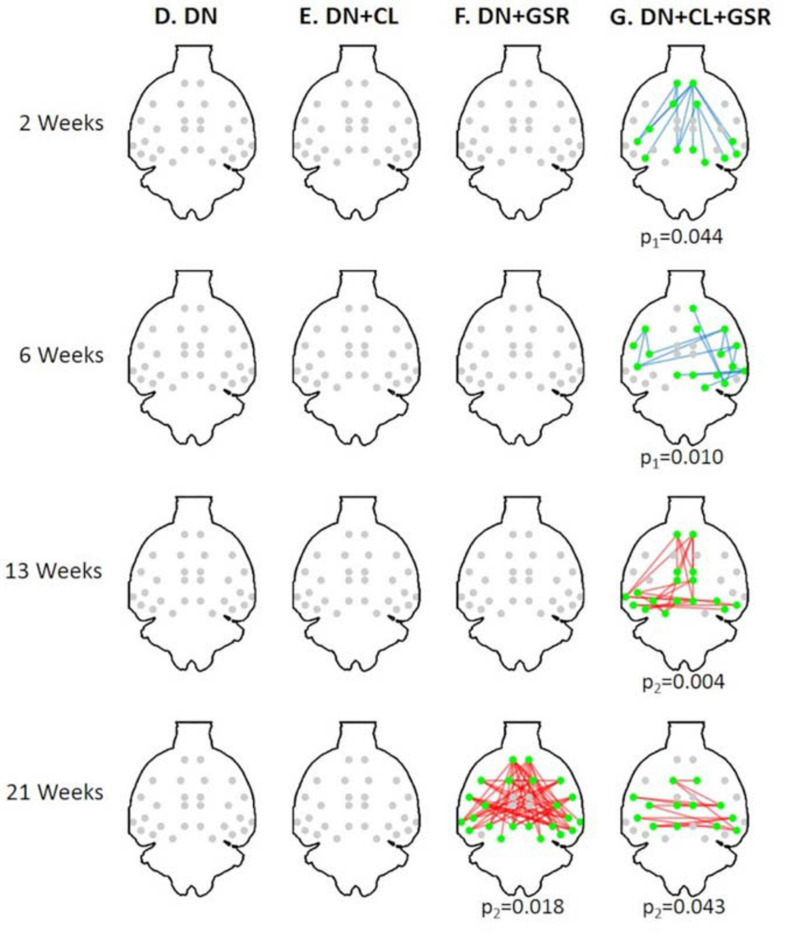
Significant differences in FC between the CTL and STZ groups of pooled Cohorts 1 + 2 at each timepoint (2, 6, 13, and 21 weeks) for data processing approaches **(D–G)** under NBS threshold 2.2. No significant difference was found with pipelines **(D–F)** (except pipeline **F** at 21 weeks). In particular, previous widespread differences at 6 weeks with pipelines **(E,F)** from Cohort 1 (Figure 8) disappeared entirely for Cohorts 1 + 2. Pipeline **(G)** exhibited similar group differences to previously (Figure 8) with the exception of new significant edges at 21 weeks, given a large increase in number of datasets at 21 weeks with the addition of Cohort 2. Blue edges indicate group differences in contrast 1 (*STZ* > *CTL*), and red edges indicate group differences in contrast 2 (*STZ* < *CTL*). *p*_1_ and *p*_2_ are FWER corrected *p*-values for contrast 1 and contrast 2, respectively. Green nodes indicate ROIs involved in group differences.

The proposed pipeline G also yielded the minimal within-group variability in control rats of Cohort 2 at all timepoints, compared with three other pipeline variants without GSR and/or cleaning, in agreement with results on Cohort 1 (Figure 11). Moreover, between-group differences in FC in the combined datasetCohorts 1 + 2 (Figure 12) were consistent with that in Cohort 1 alone (Figure 6), in a pattern of initial hyperconnectivity and later hypoconnectivity from 2 to 13 weeks. At 21 weeks, Cohort 1 alone was likely underpowered due to missing datasets, and no group differences were reported with pipeline G. The combination of Cohorts 1 + 2 enabled to reveal widespread hypoconnectivity in STZ at 21 weeks (see Supplementary Figure 6 for a detailed comparison of significant edges for Cohort 1 and Cohorts 1 + 2 at all timepoints). In contrast, group differences as highlighted by pipelines E (DN + CL) and F (DN + GSR) showed less consistency between Cohort 1 and Cohorts 1 + 2, with dramatic changes in outcome at 6 weeks particularly.

## Discussion

In this work, we proposed a novel rs-fMRI processing pipeline adapted for rat data: PIRACY, which combines MP-PCA denoising, ICA cleaning, and GSR. We built a dedicated FIX ICA classifier for rat brain that showed a high accuracy in distinguishing artifactual ICA components from the rs-fMRI signal after training. We evaluated the performance of the proposed pipeline by comparing it to six other possible approaches that excluded denoising, artifact cleaning, and/or GSR. We further evaluated the pipeline on separate datasets from an independent cohort. We showed that these three steps were essential in minimizing the within-group variability in the healthy control group. Differences between a control group and a diseased group using the STZ animal model were found more consistently across timepoints with pipeline G and were also more consistent between different analysis approaches: ROI-to-ROI FC or group-level ICA.

We included a novel thermal noise reduction method based on MP-PCA applied to rs-fMRI data that substantially improved the tSNR, resulting in fewer artifactual components to be cleaned in the ICA decomposition, and improved the statistical strength and anatomical consistency of components classified as signal. While the MP-PCA denoising technique was initially implemented for diffusion MRI data (Veraart et al., 2016), it has recently also shown great value for task fMRI (Ades-Aron et al., 2020), and it is also applicable to rs-fMRI. Indeed, resting-state neural activity may appear as random temporal events, but it falls into a specific 0.01–0.3 Hz frequency range, which makes it discernible from white Gaussian thermal noise, with a flat power spectral density. This feature is apparent in the normality test (Figure 4E) for the residuals of MP-PCA denoising. Furthermore, denoising is performed within a sliding window of 5 × 5 × 5 voxels, within which the BOLD fluctuations are likely to be correlated (cluster), whereas the thermal noise is not. Thus, the thermal noise reduction using the MP-PCA approach is very unlikely to remove the genuine BOLD fluctuations and, on the contrary, will improve the sensitivity of the analysis for the latter, as is apparent from the ICA decomposition with and without denoising (Figure 5).

It is often suggested that ICA also has denoising properties (McKeown et al., 2003; Griffanti et al., 2014), which need be clarified. On one hand, if just a few artifactual components are removed from the signal (as in FIX cleaning), the effect of ICA is primarily to remove structured noise, and not thermal (random) noise. On the other hand, if the ICA decomposition is used to keep and examine just a few ICs that appear anatomically consistent with RSNs, then indeed most of thermal noise is also removed in that process (Beckmann and Smith, 2004). However, group-level ICA suffers from its own limitations (Cole et al., 2010) and is not necessarily the appropriate analysis tool for all studies, and seed-based analyses are expected to benefit greatly from prior denoising using MP-PCA. Another study using MP-PCA denoising prior to task fMRI analysis reported an increase of 60% in SNR and improved statistics and extent of the activation (Ades-Aron et al., 2020).

Interestingly, MP-PCA denoising alone or even combining with GSR had almost no effect in reducing intra-group variability (Figure 6, columns D, F vs. A), and it also had no contribution to the detection of between-group difference in pipelines D and F compared with the variants without it (pipelines A and B, Figure 8).

Head motion during fMRI acquisitions is one of the major confounding factors that leads to artificial correlation compromising the interpretation of rs-fMRI data (Van Dijk et al., 2012; Maknojia et al., 2019). However, compared with human studies where head motion is common, rodent studies are less impacted by this confound due to the restraint achieved by a fixation setup with ear bars and a bite bar and the use of anesthesia (Pan et al., 2015). In our datasets, no apparent head motion was observed by visual inspection of time courses except for two datasets, which were discarded. Moreover, based on the framewise displacement (FD) analysis (Power et al., 2012), head motion in our rat datasets was extremely small with displacements less than 1 μm (<0.3% of voxel size) for 75% datasets and less than 7 μm (<2% of voxel size) for all (Supplementary Figure 7), whereas in human data, it is on the order of several tenths of a millimeter (∼5–10% of voxel size) (Power et al., 2012). Motion correction was, therefore, skipped in the proposed data processing pipeline since it has been shown to introduce spurious correlations (Grootoonk et al., 2000; Chuang et al., 2019; Sirmpilatze et al., 2019).

The FIX-based artifact auto-classification has already been applied in human and mouse fMRI datasets (Salimi-Khorshidi et al., 2014; Zerbi et al., 2015). In this work, this automatic artifact removal approach was for the first time implemented for rat data. After being trained in a manually classified dataset, the classifier showed a high accuracy in identifying artifact components from rs-fMRI signal in an untouched test dataset. Nonetheless, Table 2 shows that there is a trade-off between the recall and precision in the fully automatic classification of artifact components, which means that it is not possible to achieve both very high recall and precision with one FIX threshold. However, in practice, this problem could be addressed by half automated classification in which two auto-classifications are first performed with, respectively, low and high thresholds (20 and 70 for instance), and then the difference between their two artifact lists are manually examined. In this way, by manually classifying a small portion of ICA components (∼24%), we were able to achieve a very high classification accuracy in a short time. Note that the training set was cleaned aggressively in order to give flexibility in aggressiveness/conservatism for test datasets by adjusting the threshold. This classifier is available along with the rest of the pipeline code^3^. The impact of ICA cleaning alone on individual datasets suggested that this approach is not sufficient for a comprehensive mitigation of artifacts in rat rs-fMRI data (Figure 7).

Although controversial, GSR is still commonly used in the analysis of rs-fMRI data (Falahpour et al., 2018) due to its capability of reducing the effects of respiration and motion on FC estimates (Birn, 2012; Yan et al., 2013; Power et al., 2014) and enhancing the spatial specificity of positive correlations (Fox et al., 2009). Here, we found that pipelines B and F that included GSR but no CL had little effect in reducing intra-group variability compared with the baseline protocol A and could also not systematically address pronounced artifacts in individual connectivity matrices. However, combined with ICA-based cleaning, GSR reduced most within-group variability in the healthy CTL group for both Cohorts 1 and 2 and revealed differences in connectivity between the CTL and STZ groups most consistently across timepoints. The benefits of CL + GSR have previously been highlighted in human fMRI data to strengthen the association between FC and behavior (Li et al., 2019).

Taken separately, minimizing within-group variability could favor pipelines that wipe out any relevant information-carrying signal while favoring widespread between-group differences could select a pipeline with the highest false-positive rate. Satisfying both criteria concomitantly though prevents either scenario. As far as low intra-group variability being potentially a signature of over-cleaning, we underline that the average FC in the control group (Figure 9, bottom left halves of each matrix) displays expected features of strong inter-hemispheric connectivity between L/R regions as well as, for instance, anti-correlation between midline regions of the DMN and the sensorimotor system, as previously reported (Gozzi and Schwarz, 2016), which argue in favor of preserving expected baseline FC with our optimized pipeline. As mentioned, MP-PCA denoising results in more clear-cut ICA decomposition where fewer components are labeled as structural noise and removed. Over-cleaning is perhaps instead manifest in the pipeline variant without MP-PCA denoising (pipeline C: ICA cleaning + GSR) that achieved the minimal intra-group variability due to more ICs being labeled as structured noise and removed (see Figure 6). The positive influence of MP-PCA denoising on downstream ICA decomposition and identification of stronger and cleaner RSNs suggests that such an approach may also be beneficial for identifying relevant variability within a control group (Bergmann et al., 2020).

A pipeline’s ability to reveal true group differences assumes that such true differences exist, which may be challenging to ascertain. First, we underline that the proposed PIRACY pipeline revealed group differences consistently across timepoints and across integration of additional datasets (Cohort 2) to the initial Cohort 1. In contrast, other pipelines either revealed no differences at all (without CL nor GSR) or differences that were inconsistent across timepoints and across dataset pooling, and thereby indeed pointed to false positives. For example, pipeline E (DN + CL) showed overwhelming group differences only at 6 weeks (Cohort 1), which disappeared in Cohort 1 + 2 analysis, and nothing otherwise. Pipeline F (DN + GSR) showed no differences at 2 and 13 weeks and overwhelming group differences at 6 and 21 weeks (Cohort 1), with only the latter being sustained in the Cohort 1 + 2 analysis.

In terms of the reliability of the differences pointed by the PIRACY pipeline, while false positives cannot be excluded due to the relatively small sample size, we stress that group differences were indeed expected at all chosen timepoints and the pattern that PIRACY showcased for these changes agreed with previous literature on this animal model assessed from histology, behavior, and volumetry (Lester-Coll et al., 2006; Kraska et al., 2012; Knezovic et al., 2015). The FC analysis resulting from Cohort 1 and its pertinence with respect to the animal model and to AD have been published separately (Tristão Pereira et al., 2021). Briefly, STZ rats in Cohort 1 exhibited not only altered FC but also intra-axonal damage and demyelination (assessed using diffusion MRI) in brain regions typical of AD, in a temporal pattern of acute injury, transient recovery/compensation, and chronic degeneration. The non-monotonic pattern in FC changes was characterized by initial hyper-connectivity and impaired network dissociation, followed by later hypo-connectivity, consistent with patterns found in pre-clinical AD and mild cognitive impairment (Dickerson et al., 2005; Schultz et al., 2017). The switch occurred between the 6- and 13-week timepoints. FC in the CTL group was consistent with previous reports that midline regions of the DMN are anti-correlated with the sensorimotor system (Gozzi and Schwarz, 2016). In STZ animals, these anti-correlations between DMN and LCN were initially reduced (2 and 6 weeks), suggesting less efficient network dissociation and brain processing (Fox et al., 2005). Concomitantly, hyperconnectivity within the DMN was found in STZ rats at these early timepoints. By 13 weeks, however, STZ rats exhibited reduced connectivity (or hypoconnectivity) in regions typically involved in AD, also consistent with eventual memory impairment in this animal model and reduced FC in AD patients (Binnewijzend et al., 2012; Franzmeier et al., 2019). While Cohort 1 had too few datasets at 21 weeks to identify group differences, the combination of Cohorts 1 + 2 in the context of the current work revealed patterns of sustained inter-hemispheric hypoconnectivity particularly between posterior brain and DMN regions at this timepoint. In the context of sustained glucose hypometabolism, these non-monotonic trends–also reported in behavioral studies of this animal model (Knezovic et al., 2015) as well as in human AD (Dickerson et al., 2005; Fortea et al., 2011, 2014; Pegueroles et al., 2017; Sierra-Marcos, 2017; Dong et al., 2020)–suggest a compensatory mechanism, possibly recruiting ketone bodies, that allows a partial and temporary repair of brain structure and function.

### Limitations

First, free breathing of animals was chosen as an easier setup, best suited for longitudinal studies. While it certainly brings in higher respiratory variations to fMRI data than artificial ventilation, the breathing rate was carefully monitored and was relatively stable during the 10′ of fMRI acquisition. Breathing fluctuations were further mitigated at the level of ICA cleaning and GSR. Second, animals breathed a consistent mixture of air and oxygen, but SpO_2_ was not monitored. While we did maintain a strictly consistent experimental protocol throughout the study in terms of gas mixture, anesthesia levels, and timings, varying SpO_2_ levels might confound the BOLD signal and thereby the results. We note, however, that a recent meta-analysis has shown that, even when blood gas levels are monitored, their impact on the BOLD signal is not consistent/systematic across studies (Steiner et al., 2020), which makes it difficult to account for. Finally, how our findings generalize to completely different datasets, particularly in terms of disease model, remains to be investigated.

## Conclusion

We conclude that the PIRACY processing pipeline for rat rs-fMRI data proposed herein, which includes MP-PCA denoising, a FIX auto-classification and cleaning of structured artifacts uncovered by ICA, and GSR, allowed to greatly reduce the within-group variability and improve the detection of between-group differences at the same time. This data processing pipeline, therefore, has strong potential to improve the sensitivity and reproducibility of rs-fMRI studies on rat models of disease and injury.

## Data Availability Statement

The pipeline code and FIX rat classifier are available at https://github.com/Mic-map/PIRACY. The data pertaining to Cohort 1 is available at https://openneuro.org/datasets/ds003520.

## Ethics Statement

The animal study was reviewed and approved by the Cantonal Service for Veterinary Affairs (Vaud, CH).

## Author Contributions

IJ and RG conceived and designed the experiments. YD performed the experiments. YD and TY analyzed the data. All authors wrote the manuscript.

## Conflict of Interest

The authors declare that the research was conducted in the absence of any commercial or financial relationships that could be construed as a potential conflict of interest.

## Footnotes

1 https://fsl.fmrib.ox.ac.uk/fsl

2 https://www.nitrc.org/projects/whs-sd-atlas

3 https://github.com/Mic-map/PIRACY
