# Supplementary material for: PIRACY: An Optimized Pipeline for Functional Connectivity Analysis in the Rat Brain
Source: Front Neurosci. 2021 Mar 26;15:602170. doi: 10.3389/fnins.2021.602170 (PMC8032956; doi:10.3389/fnins.2021.602170)
Supplement: Supplementary file 1 [file Image_1.PDF]

Supplementary material

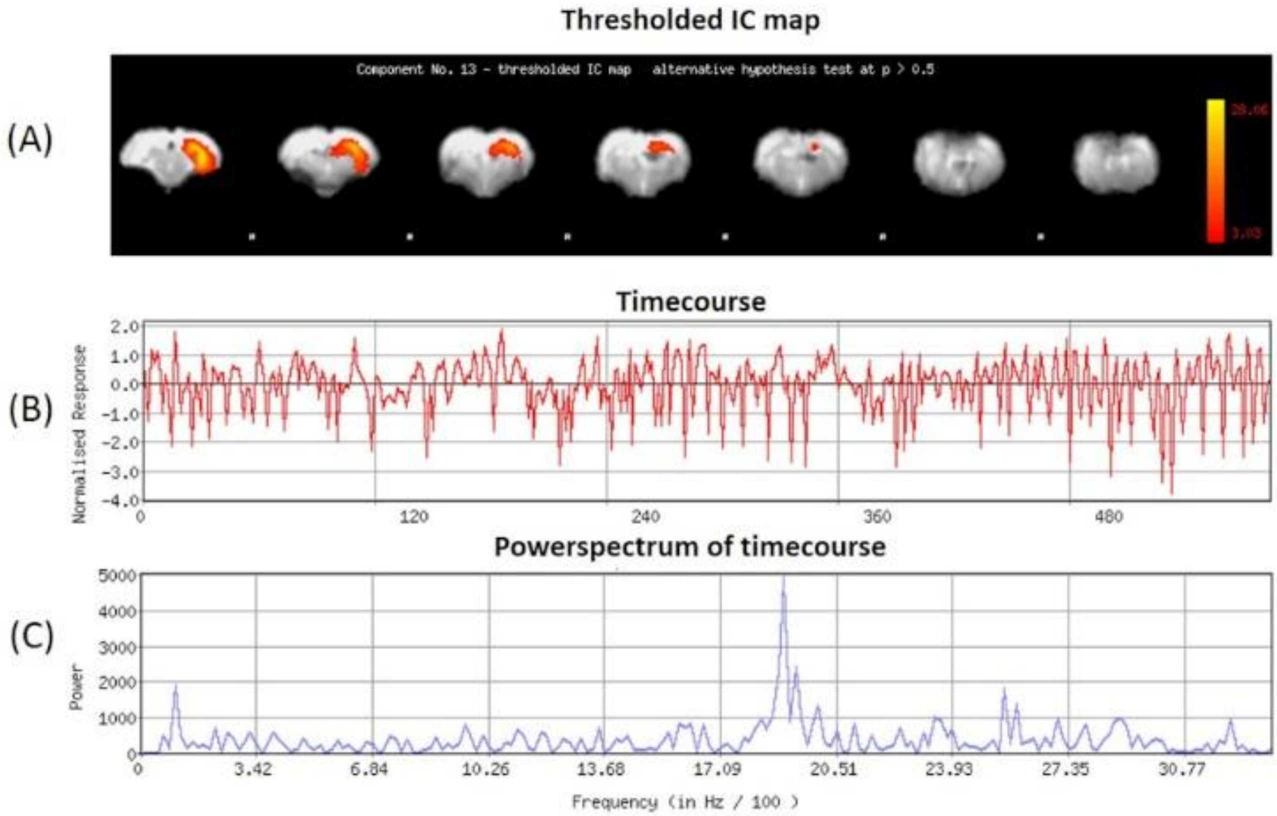

Supplementary Figure 1. Example of independent component which is anatomically consistent (left hippocampus) yet corrupted by breathing: thresholded spatial map (A), timecourse (B) and the power spectrum of timecourse (C). The power spectrum shows an aliased frequency with a peak at around 0.18 Hz due to breathing.

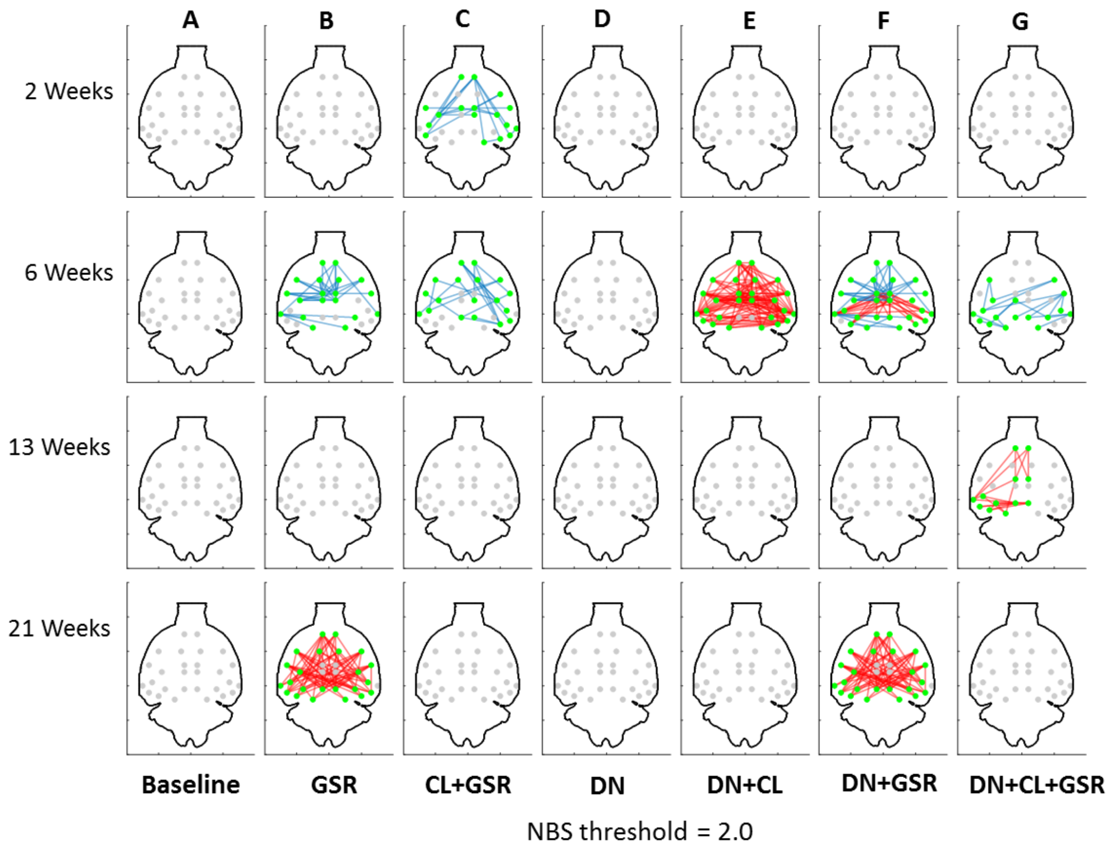

Supplementary Figure 2. The significant difference in functional connectivity between CTL and STZ groups at each timepoint for each data processing approach under NBS threshold of 2.0. A: baseline (SC+SM+HP), B: GSR, C: CL+GSR, D: DN, E: DN +CL, F: DN +GSR, G: DN +CL+GSR. Blue edges indicate group differences in contrast1 (STZ>CTL) and red edges indicate group differences in contrast2 (STZ<CTL).

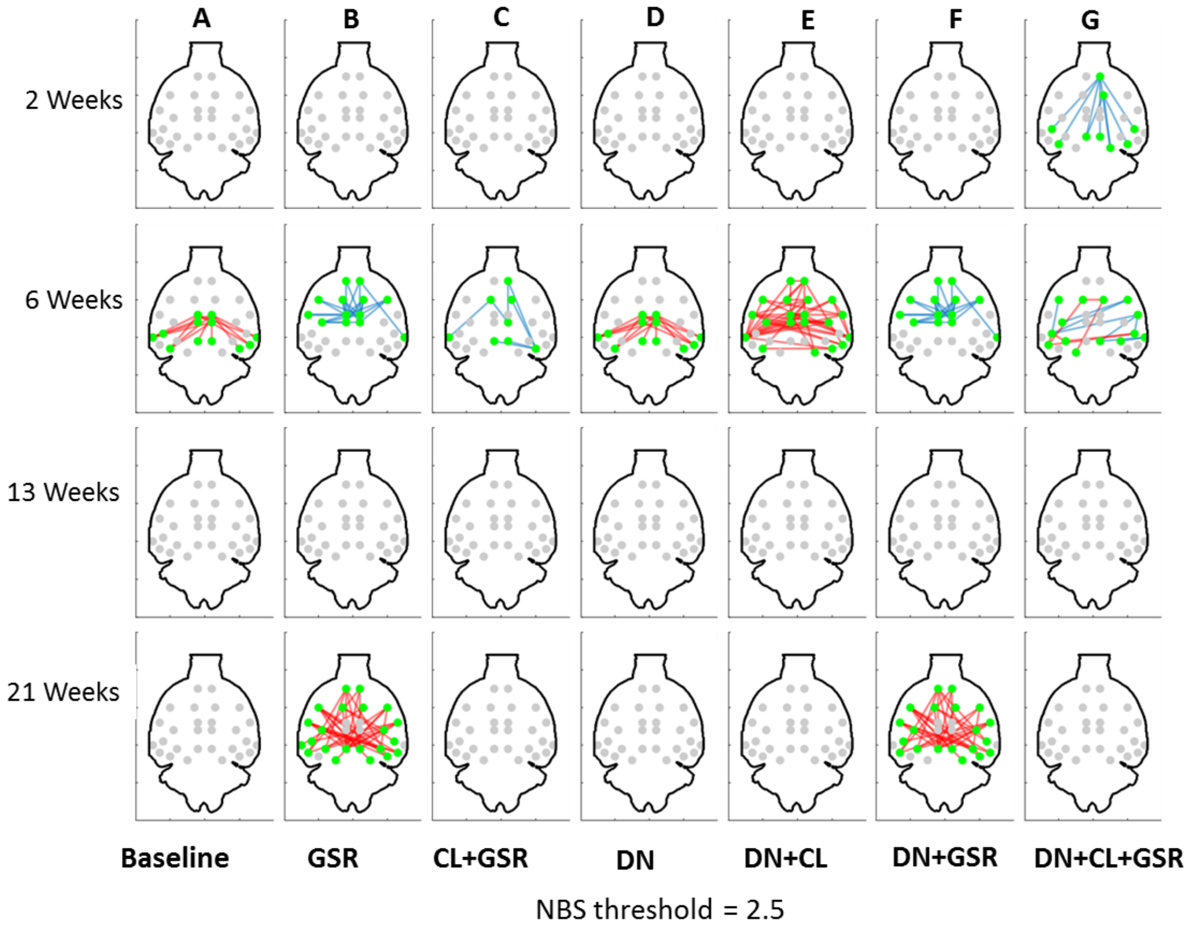

Supplementary Figure 3. The significant difference in functional connectivity between CTL and STZ groups at each timepoint for each data processing approach under NBS threshold of 2.5. A: baseline (SC+SM+HP), B: GSR, C: CL+GSR, D: DN, E: DN +CL, F: DN +GSR, G: DN +CL+GSR. Blue edges indicate group differences in contrast1 (STZ>CTL) and red edges indicate group differences in contrast2 (STZ<CTL).

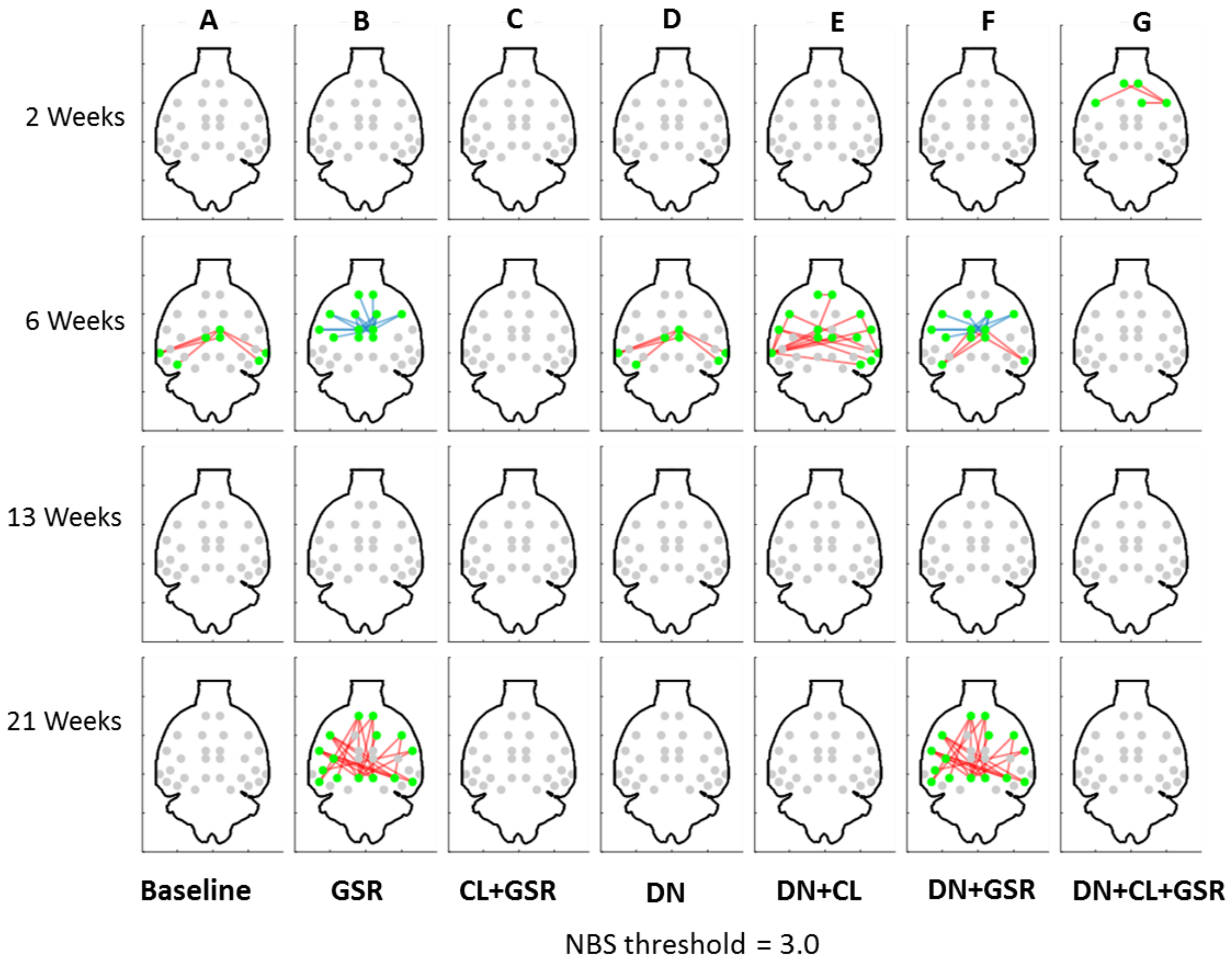

Supplementary Figure 4. The significant difference in functional connectivity between CTL and STZ groups at each timepoint for each data processing approach under NBS threshold of 3.0. A: baseline (SC+SM+HP), B: GSR, C: CL+GSR, D: DN, E: DN +CL, F: DN +GSR, G: DN +CL+GSR. Blue edges indicate group differences in contrast1 (STZ>CTL) and red edges indicate group differences in contrast2 (STZ<CTL).

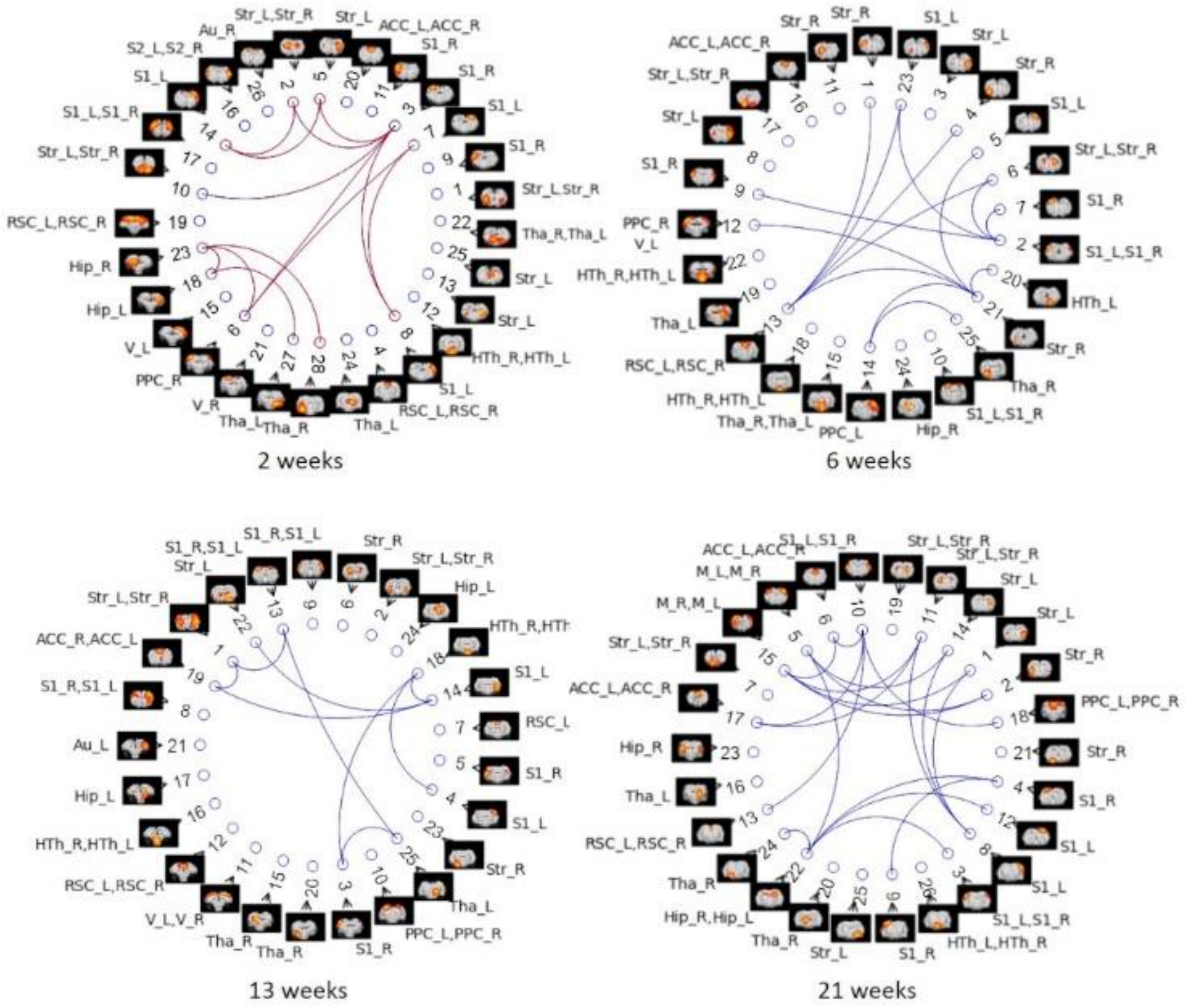

Supplementary Figure 5. Between-group differences in ICA-based functional connectivity without GSR (full correlation) at NBS threshold of 2.2 for each timepoint in Cohort 1. Colored edges display the existence of STZ-CTL difference in connections between IC's. Each IC is denoted by a spatial map and its IC number. The nodes of IC's are listed in an order based on its position in brain (anterior to posterior). ROI labels are attached to every ICA component. Artefactual components were removed and the IC number was reordered accordingly. Blue edges indicate group differences in contrast1 (*STZ* > *CTL*) and red edges indicate group differences in contrast2 (*STZ* < *CTL*).

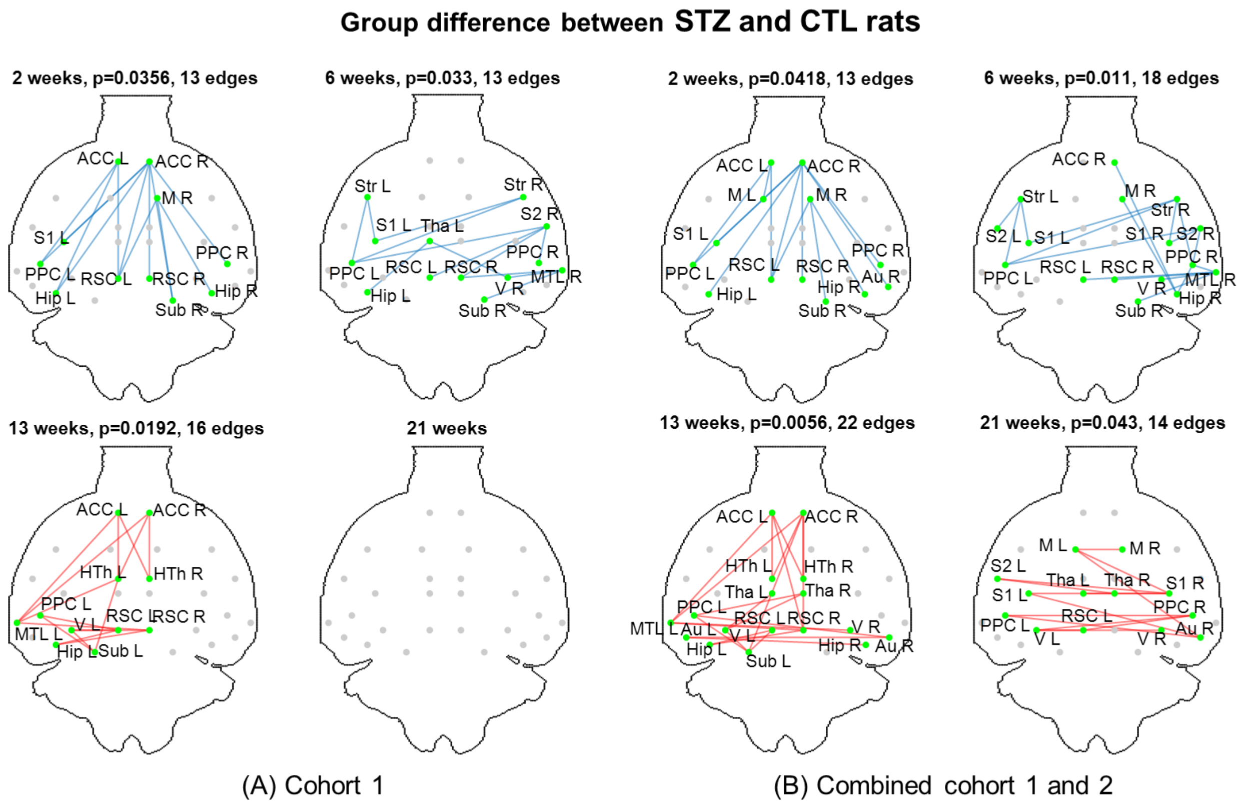

Supplementary Figure 6. Significant differences in FC between CTL and STZ groups in cohort 1 (A) and combined cohort 1 and 2 (B) processed using the PIRACY pipeline G displayed in graph networks at 4 timepoints. Blue/red edges and green nodes indicate connections with significant difference. The ***p***-value (< ***0.05***) for each network was given after FWER correction at NBS threshold of 2.2. Blue edges indicate group differences in contrast1 (***STZ*** > ***CTL***) and red edges indicate group differences in contrast2 (***STZ*** < ***CTL***).

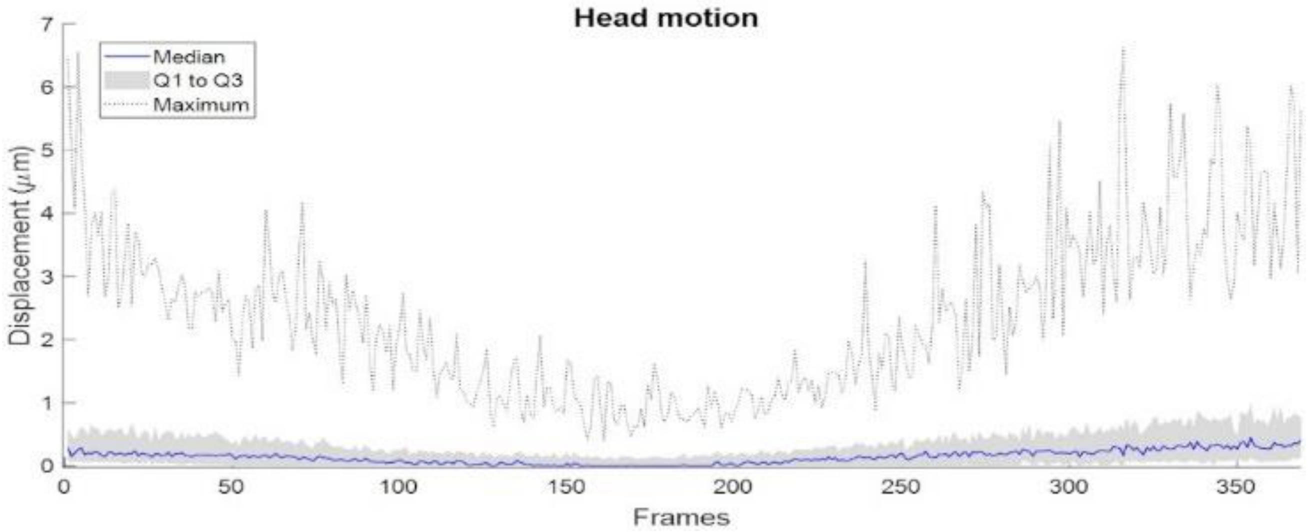

Supplementary Figure 7. Head motion evaluated in terms of framewise displacement (μm). The blue curve indicates median FD in the datasets along all time frames. The gray shade is the FD ranging from 25 to 75 percentiles in the datasets. The maximum displacements are illustrated in the black dashed line. FD is less than 1 μm (< 0.3% of voxel size) for 75% datasets and less than 7 μm (< 2% of voxel size) for all. Here, FD was calculated at a 5 mm radius, a tenth of the radius used in human data (Power et al., 2012).

Supplementary Table 1. Connections (edges) with significant between-group differences in ROI-based functional connectivity of pipeline G (NBS threshold=2.2). This table underlies Figure 10 (B). *ACC: anterior cingulate cortex; RSC: retrosplenial cortex; PPC: posterior parietal cortex; MTL: medial temporal lobe; Hip: hippocampus; Sub: subiculum; Au: auditory; V: visual; S1/S2: primary/secondary somatosensory; M: motor; Str: striatum; Tha: thalamus; HTh: hypothalamus. L/R: left/right.*

Time points	Connections		Correlation coefficients		contrast
	Node A	Node B	STZ	CTL	
2 weeks	ACC_L	RSC_L	0.43	0.27	STZ > CTL
	ACC_R	RSC_L	0.39	0.14	
	ACC_R	RSC_R	0.38	0.15	
	ACC_L	PPC_L	0.09	-0.09	
	ACC_R	PPC_L	0.07	-0.18	
	ACC_R	PPC_R	0.11	-0.14	
	ACC_L	Hip_L	0.04	-0.12	
	ACC_R	Hip_L	0.00	-0.19	
	ACC_R	Sub_R	0.04	-0.13	
	ACC_R	S1_L	-0.11	-0.30	
	RSC_L	M_R	0.14	-0.05	
	Hip_R	M_R	-0.04	-0.22	
	Sub_R	M_R	0.01	-0.19	
6 weeks	RSC_R	MTL_R	0.10	-0.07	STZ > CTL
	MTL_R	Sub_R	0.13	-0.08	
	MTL_R	V_R	0.27	-0.01	
	RSC_L	S2_R	-0.10	-0.26	
	RSC_R	S2_R	-0.05	-0.24	
	PPC_L	S2_R	-0.12	-0.31	
	PPC_R	S2_R	0.04	-0.20	
	PPC_L	Str_L	-0.27	-0.45	
	S1_L	Str_L	-0.10	-0.34	
	PPC_L	Str_R	-0.26	-0.52	
	S1_L	Str_R	-0.14	-0.44	
	Hip_L	Tha_L	0.39	0.22	
	V_R	Tha_L	-0.03	-0.20	
13 weeks	RSC_L	PPC_L	0.09	0.33	STZ < CTL
	ACC_L	MTL_L	-0.15	0.06	
	ACC_R	MTL_L	-0.11	0.04	
	RSC_R	MTL_L	-0.09	0.11	
	RSC_L	Hip_L	0.07	0.32	
	RSC_R	Hip_L	0.02	0.23	
	PPC_L	Sub_L	-0.22	0.09	
	RSC_L	V_L	0.26	0.54	
	RSC_R	V_L	0.18	0.44	
	Sub_L	V_L	-0.03	0.34	
	ACC_L	HTh_L	-0.09	0.09	
	ACC_R	HTh_L	-0.09	0.09	
	MTL_L	HTh_L	-0.10	0.14	
	Sub_L	HTh_L	-0.09	0.16	
	ACC_L	HTh_R	-0.10	0.06	
	ACC_R	HTh_R	-0.13	0.06	

Supplementary Table 2. Connections (edges) with significant between-group differences in group ICA-based functional connectivity with GSR (NBS threshold=2.2). Each node is an ICA component and each component covers one or more anatomical ROIs. This table underlies Figure 11. *ACC: anterior cingulate cortex; RSC: retrosplenial cortex; PPC: posterior parietal cortex; MTL: medial temporal lobe; Hip: hippocampus; Sub: subiculum; Au: auditory; V: visual; S1/S2: primary/secondary somatosensory; M: motor; Str: striatum; Tha: thalamus; HTh: hypothalamus. L/R: left/right.*

Time points	Connections		ROI labels of nodes		Correlation coefficients		contrast
	Node A	Node B	A	B	STZ	CTL	
2 weeks	6	7	PPC_R	S1_L	0.19	0.36	STZ < CTL
	3	8	S1_R	S1_L	0.05	0.22	
	7	8	S1_L	S1_L	0.15	0.43	
	3	10	S1_R	Str_L,Str_R	-0.28	-0.01	
	1	19	Str_L,Str_R	RSC_L,RSC_R	-0.27	-0.13	
	6	23	PPC_R	Hip_R	-0.06	0.20	
	18	23	Hip_L	Hip_R	0.15	0.51	
	21	23	V_R	Hip_R	0.00	0.28	
	18	24	Hip_L	Tha_L	0.14	0.42	
	21	24	V_R	Tha_L	0.04	0.23	
	23	24	Hip_R	Tha_L	0.12	0.39	
	15	27	V_L	Tha_L	-0.17	0.09	
	18	27	Hip_L	Tha_L	-0.16	0.22	
	19	27	RSC_L,RSC_R	Tha_L	-0.38	-0.16	
	24	27	Tha_L	Tha_L	-0.17	0.05	
	26	27	Au_R	Tha_L	-0.16	0.05	
6 weeks	1	12	Str_R	PPC_R	-0.14	-0.30	STZ > CTL
	5	21	S1_L	Str_L,Str_R	-0.21	-0.42	
	12	21	PPC_R	Str_L,Str_R	-0.08	-0.37	
	13	21	RSC_L,RSC_R	Str_L,Str_R	-0.07	-0.23	
	14	21	PPC_L	Str_L,Str_R	-0.07	-0.37	
	13	23	RSC_L,RSC_R	S1_L	0.08	-0.12	
	14	25	PPC_L	Tha_R	-0.09	-0.31	
	5	12	S1_L	PPC_R	-0.10	0.19	STZ < CTL
	8	12	Str_L	PPC_R	-0.22	-0.04	
	2	13	S1_L,S1_R	RSC_L,RSC_R	-0.17	0.08	
	12	13	PPC_R	RSC_L,RSC_R	0.01	0.15	
	2	14	S1_L,S1_R	PPC_L	0.00	0.23	
	10	14	S1_L,S1_R	PPC_L	-0.07	0.15	
	11	14	Str_R	PPC_L	-0.21	-0.05	
	12	20	PPC_R	HTh_L	-0.12	0.03	
	2	24	S1_L,S1_R	Hip_R	-0.19	-0.05	
13 weeks	9	15	S1_R,S1_L	Tha_R	-0.16	0.02	STZ < CTL
	9	16	S1_R,S1_L	HTh_R,HTh_L	-0.02	0.18	
	9	18	S1_R,S1_L	HTh_R,HTh_L	-0.15	0.14	
	15	18	Tha_R	HTh_R,HTh_L	-0.17	0.14	
	16	22	HTh_R,HTh_L	Tha_R	-0.02	0.20	
	18	24	HTh_R,HTh_L	Tha_L	-0.27	0.00	
	22	24	Tha_R	Tha_L	-0.10	0.14	
